# Correlation between improvement in visual acuity and QOL after Ranibizumab treatment for age-related macular degeneration patients: QUATRO study

**DOI:** 10.1186/s12886-021-01816-7

**Published:** 2021-01-23

**Authors:** Yuji Oshima, Yumi Ishibashi, Naoyasu Umeda, Tatsuo Nagata, Shigeo Yoshida, Eiichi Uchio, Hiroyuki Kondo, Koh-hei Sonoda, Tatsuro Ishibashi

**Affiliations:** 1 Department of Ophthalmology, Graduate School of Medical Sciences, Kyushu University, 3-1-1 Maidashi, Higashi-ku, Fukuoka, 812-8582 Japan; 2 Department of Ophthalmology, Kurume University School of Medicine, Fukuoka, Japan; 3 Department of Ophthalmology, School of Medicine, Fukuoka University, Fukuoka, Japan; 4 Department of Ophthalmology, University of Occupational and Environmental Health, Fukuoka, Japan

**Keywords:** Age-related macular degeneration, Patient satisfaction, Quality of vision, Ranibizumab

## Abstract

**Background:**

To evaluate the correlation between visual acuity improvement and vision-related QOL after ranibizumab treatment in Japanese patients with AMD.

**Methods:**

In this one-year prospective, interventional, open-label, multicenter study involving four sites, patients with neovascular AMD were enrolled and observed for 12 months. Treatment-naïve patients received 0.5 mg ranibizumab as needed after three initial monthly doses. The best corrected visual acuity (BCVA) and central macular thickness (CMT) were measured at every visit. Evaluations with the 25-item National Eye Institute Visual Function Questionnaire (NEI-VFQ-25) and patient satisfaction questionnaire were performed at baseline and 3 and 12 months after initial treatment. The primary endpoint was change in BCVA and QOL 3 months after ranibizumab treatment. QOL outcomes were also assessed in the better and poor BVCA subgroups.

**Results:**

The study enrolled 100 patients. The mean logMAR BCVA after treatment improved significantly from 0.43 to 0.30 at 3 months (*p*< 0.0001), and 0.28 at 12 months (*p*< 0.0001). The mean NEI-VFQ-25 composite scores improved from 79.48 to 84.13 at 3 months (*p*< 0.0001), and 86.0 at 12 months (*p*< 0.0001). The 3 and 12-month changes in NEI-VFQ-25 score and BCVA showed significant correlation. In the poor baseline visual acuity group (decimal BCVA ≤0.5), there was a significant correlation between the changes in the NEI-VFQ-25 score and BCVA (*p*=0.02) but not in the better baseline visual acuity group (decimal BCVA > 0.6, *p*=0.1) at 3 months. There were no significant differences in the satisfaction questionnaire score from baseline to at 3 months (*p*=0.54) and 12 months (*p*=0.23). The average CMT improved significantly from 340 to 264 μm at 3 months (*p*< 0.0001) and to 268 μm at 12 months (p< 0.0001).

**Conclusions:**

Intravitreal ranibizumab treatment resulted in improvement in visual acuity, anatomical change, and visual function change in Japanese AMD patients. Significant improvement was seen in patient visual function, and this was correlated with changes in VA, except immediately after loading dose treatment in patients with higher baseline VA. The patients’ satisfaction with the treatment remained unchanged during the study period.

**Trial registration:**

This study is registered at UMIN Clinical Trials Registry (UMIN000012013). Registered October 10, 2013, as prospective study.

## Key messages

1. Anti-VEGF therapy is known to be anatomically and functionally beneficial for patients with neovascular AMD.
2. Improvements in visual acuity, anatomical change, and visual function change were reported in Japanese patients with nAMD following intravitreous ranibizumab treatment.
3. Significant improvements in quality of vision were seen in both patients with poor and better baseline visual acuity.
4. Patients’ satisfaction toward the treatment remained unchanged throughout the duration of the study.

## Background

Age-related macular degeneration (AMD) is a significant cause of blindness in elderly populations, particularly in more economically developed countries. AMD is classified as either neovascular or atrophic based on the presence or lack of choroidal neovascularization (CNV) beneath the retina, respectively [1]. The exact pathogenesis of AMD is still unclear, but the complement cascade, metabolism, some environmental factors, remodeling of the retinal extracellular collagen matrix, the angiogenesis pathway, and lipid transport are thought to promote its development [2].

Anti-vascular endothelial growth factor (VEGF) agents are commonly used for treatment and recognized to improve vision in neovascular AMD (nAMD) patients worldwide, as shown in international studies [3–5]. Japanese guidelines also recommend anti-VEGF agents as the first choice in nAMD [6]. Ranibizumab is an anti-VEGF drug and a humanized monoclonal antibody fragment, which blocks VEGF by binding it. Ranibizumab can not only avert vision loss in most patients but even culminate in significant visual gain in roughly one-third of patients. Routine monthly injections of anti-VEGF agents have been widely used as the gold standard treatment for nAMD [3, 7, 8]. Previous large-scale clinical trials for anti-VEGF therapy in nAMD patients showed that monthly injections of either ranibizumab or aflibercept improved visual acuity (VA) [3, 4, 7–11]. However, the more recent SEVEN-UP STUDY showed that a switch to “as needed” (pro re nata [PRN]) dosing regimens after monthly ranibizumab injections in the MARINA and ANCHOR studies caused eventual visual loss [12]. Therefore, many investigators prefer proactive treatment with treat-and-extend or fixed dosing regimens instead of as needed treatment to maintain the vision gain achieved after the induction treatment [13–15].

The National Eye Institute Visual Function Questionnaire 25 (NEI-VFQ-25) was developed to allow subjective assessment of vision-related function. The NEI-VFQ-25 has been validated in patients with many ocular disorders, including AMD [16, 17]. Many clinical studies have shown the effectiveness of anti-VEGF therapy for patients with nAMD, not only in terms of VA, but also vision-related quality of life both in clinical trials and in real-world assessments [18–22]. Bressler et al. examined the effects of ranibizumab on the quality of vision in nAMD patients by classifying whether the study eye was the better- or worse-seeing eye in the MARINA and ANCHOR clinical trials, and they reported improved vision-related function regardless of whether the treated eye was the better- or worse-seeing eye at baseline [22]. To our knowledge, no multicenter studies have assessed ranibizumab treatment using the NEI-VFQ-25 in Japanese patients with nAMD in a real-world setting. Herein, we report the outcomes of vision-related quality of life for Japanese patients with nAMD according to their baseline VA.

## Methods

### Study design

The QUATRO study (UMIN000012013) was a prospective, multicenter, open-label study of patients with nAMD conducted at four Japanese university hospitals between January 2014 and December 2016. The protocol was approved by the Institutional Review Board/Ethics Committee of each participating hospital and the Clinical Research Network Fukuoka Certified Review Board. All study conduct adhered to the tenets of the Declaration of Helsinki, and all participants provided written informed consent to participate in the study.

### Inclusion and exclusion criteria

Patients were diagnosed with nAMD based on comprehensive ophthalmic examinations, which we have previously published [23, 24]. Briefly, all patients underwent ophthalmic evaluations, including fundus examination, slit-lamp biomicroscopy, and decimal BCVA. Imaging examinations including angiography with fluorescein (FA) and indocyanine green (ICGA), fundus photography, and spectral-domain optical coherence tomography (OCT) were also performed. Eligible patients were aged 50 years or older and met the following inclusion criteria for the study eye: (1) decimal BCVA greater than 0.05; (2) exudative subfoveal lesion identified by OCT, ICGA, or FA. The treated eye was considered as the study eye. If both eyes were affected with nAMD, the worse-seeing eye was treated and included. The same exclusion criteria of our previously published study was used for this study (the QUATRO study) [23]. Briefly,: (1) total area of pathological lesion greater than 12 disc areas; (2) presence of non-fibrotic or fibrotic scarring constituting > 50% of the pathological lesion; (3) presence of retinal pigmented epithelium (RPE) tear, (4) history of stage 3 to 5 macular hole; (5) history of treatment with intravitreal or sub-Tenon’s injection of triamcinolone acetonide within the previous 6 months; (4) history of intraocular surgery (including cataract surgery) within the previous 3 months; (5) history of previous vitrectomy or sub-macular surgery; (6) active infection inside or around either eye; (7) history of uveitis in either eye; (8) known allergy to fluorescein, indocyanine green, or iodine, (8) pregnancy, possibility of pregnancy, or nursing; and (9) any other conditions that were regarded as unsuitable for this study by the investigators.

### Treatment and assessment schedule

**Fig. 1:**
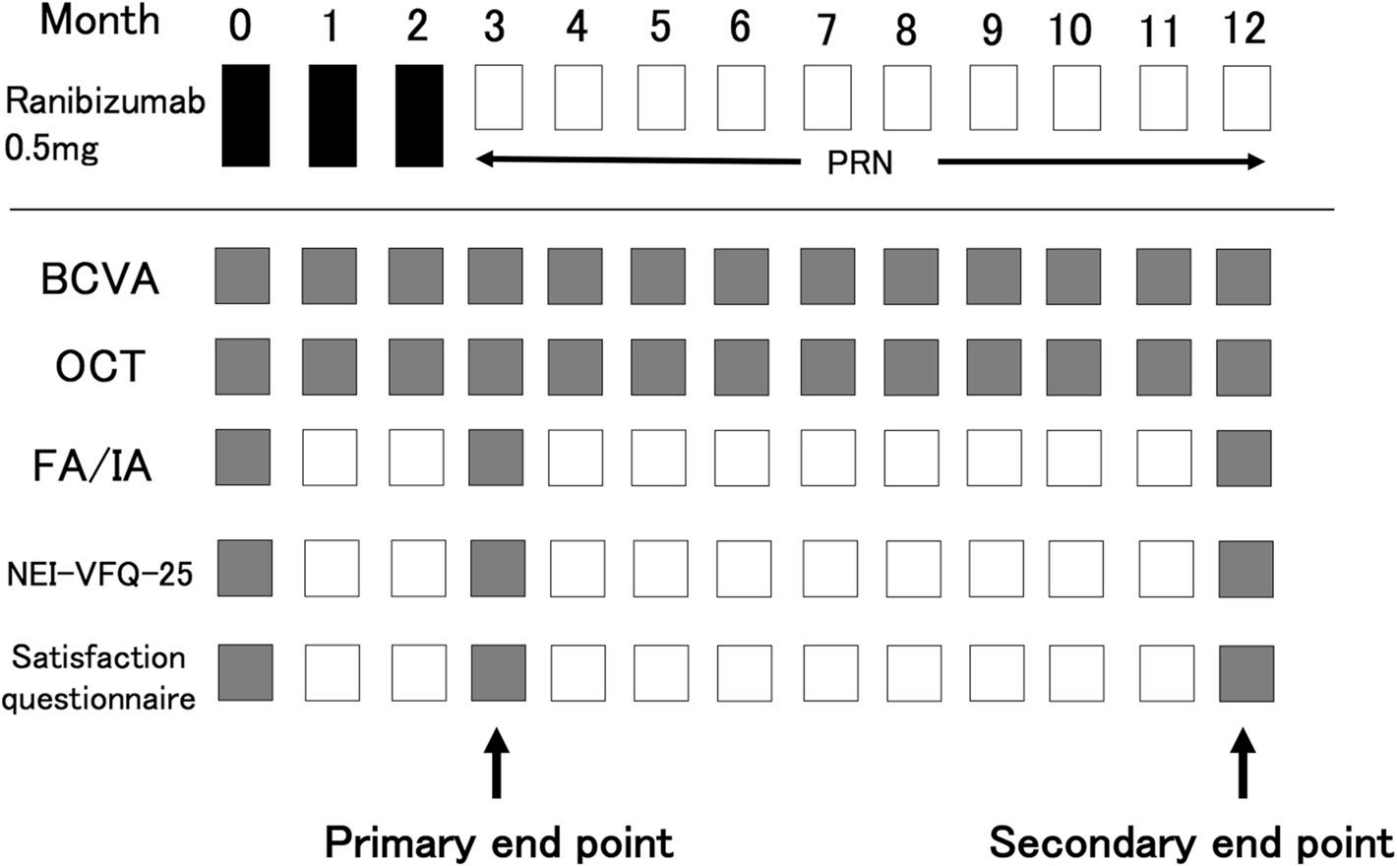
Study schedule. Treatment-naïve neovascular age-related macular degeneration (nAMD) patients received intravitreal injections of ranibizumab (IVR) (0.5 mg) as needed with monthly monitoring after three initial monthly doses. BCVA = best-corrected visual acuity. OCT = optical coherence tomography. FA = fluorescein angiography. IA = indocyanine green angiography. VFQ-25 = The 25-item National Eye Institute Visual Function Questionnaire

Intravitreal ranibizumab (0.5 mg) was administered in three loading doses at months 0, 1, and 2, followed by further injections performed as needed during monthly monitoring in months 4 to 12. The indication for additional treatment was decided according to the maintenance period guideline for intravitreal administration of ranibizumab established by the Japanese Society of Ophthalmology. Decimal BCVA was measured at each visit, using a Landolt chart, and was expressed as the logarithm of the minimal angle of resolution (logMAR). Prior to injections, color fundus photography, slit-lamp biomicroscopy, and spectral-domain OCT were performed. Central macular thickness (CMT) was determined using OCT as the average thickness of the central 1-mm thickness map measurement area. FA and ICGA images were both obtained at baseline, 3 months, and 12 months. Assessments with the 25-item National Eye Institute Visual Function Questionnaire (NEI-VFQ-25) and patient satisfaction questionnaire were performed at baseline and 3 and 12 months after initial treatment. Patient satisfaction questionnaire assessments were performed with a Likert scale model consisting of five ratings addressing the following aspects: (1) the burden of frequent visits and treatment, (2) anxiety associated with intravitreal injection (two questions), (3) anxiety associated with the results of examinations, and (4) effect of the treatment on mental health (Fig. 1).

### Outcome measurement

The primary outcome was the amount of change from baseline in BCVA (logMAR) and QOL (NEI-VFQ-25 score) 3 months after ranibizumab administration. Secondary outcome measures included the mean change in BCVA and NEI-VFQ-25 score from baseline to 12 months, mean change in CMT from baseline to 3 and 12 months, proportion of patients with exudation (intraretinal/subretinal fluid, serous retinal pigment epithelium detachment), mean change in greatest linear dimension (GLD) from baseline to 3 and 12 months, and mean change in patient satisfaction questionnaire score from baseline to 3 and 12 months. Outcomes were also assessed in the subgroups. Baseline VA was defined as poor (≤0.5 decimal VA, 20/40 Snellen) or better (decimal VA > 0.5). The impact of baseline VA (> 0.5 or ≤0.5 decimal VA) on the NEI-VFQ-25 assessments at 3 and 12 months was determined.

### Statistical analysis

Statistical analysis was performed using SAS software (version 9.3, Tokyo, Japan). In the analysis of the time course of continuous variables, the mean value of changes at each time point and their 95% confidence intervals were estimated using a linear mixed model. Estimation was performed by the maximum likelihood method; the degree of freedom was calculated by the Satterthwaite equation. In the analysis of binary data, the proportion and its 95% confidence interval at each time point were obtained by the Wilson method. Spearman’s rank correlation coefficient was used. In all analyses, *p* < 0.05 (two-sided) was considered to be statistically significant.

## Results

### Baseline characteristics

The baseline characteristics of the participants are shown in Table 1, and the backgrounds by baseline visual acuity subgroup are shown in Table 2.

**Table 1:** Baseline Characteristics

		*N*=99
Sex	Male	77 (78%)
	Female	22 (23%)
Age		73.2
BCVA (logMAR)		0.49 (0.43)
Medical history	Diabetes	20 (20%)
	Hypertension	49 (50%)
Disease duration (years)		0.5 (±0.92)
Type of AMD	typical AMD	83 (84%)
	PCV	13 (13%)
	RAP	3 (3%)
FA classification	Predominantly classic	24 (24%)
	Minimally classic	17 (17%)
	Occult	58 (59%)
Past history	None	97 (98%)
	Photocoagulation	1 (1%)
	PDT	0 (0%)
	Other anti-VEGF drugs	0 (0%)
	Others	1 (1%)

*AMD* age-related macular degeneration, *BCVA* best corrected visual acuity, *logMAR* logarithm of the minimal angle of resolution, *PCV* polypoidal choroidal vasculopathy, *RAP* retinal angiomatous proliferation, *VEGF* vascular endothelial growth factor

**Table 2:** Baseline characteristics based on baseline visual acuity

	Decimal Visual Acuity	
	Below 0.5	Above 0.6
	*N* = 58	*N* = 41
Age	74.5 (±7.9)	71.3 (±10.3)
Sex		
Male	45 (77.6%)	32 (78%)
Female	13 (22.4%)	9 (22%)
Type of AMD		
AMD	51 (87.9%)	32 (78%)
PCV	4 (6.9%)	9 (22%)
RAP	3 (5.2%)	.

*AMD* age-related macular degeneration, *PCV* polypoidal choroidal vasculopathy, *RAP* retinal angiomatous proliferation

A total of 100 patients with nAMD were enrolled in the study. Of these, one patient was judged to be ineligible by the investigator after enrollment. Thus, data from 99 patients were used in this study (77 males, 22 females); their mean age was 73.2 ± 9.1 years (range, 50–88 years). Mean BCVA was 0.43 logMAR ± 0.36, mean GLD was 3052 ± 1541 μm, and mean CMT by OCT was 340 ± 118 μm. The AMD type was classified as typical AMD in 83 eyes, polypoidal choroidal vasculopathy (PCV) in 13 eyes, and retinal angiomatous proliferation (RAP) in 3 eyes. Twenty (20%) patients had diabetes and 49 (50%) had hypertension. Average disease duration was 0.5 ± 0.92 years.

### Visual outcome

**Fig. 2:**
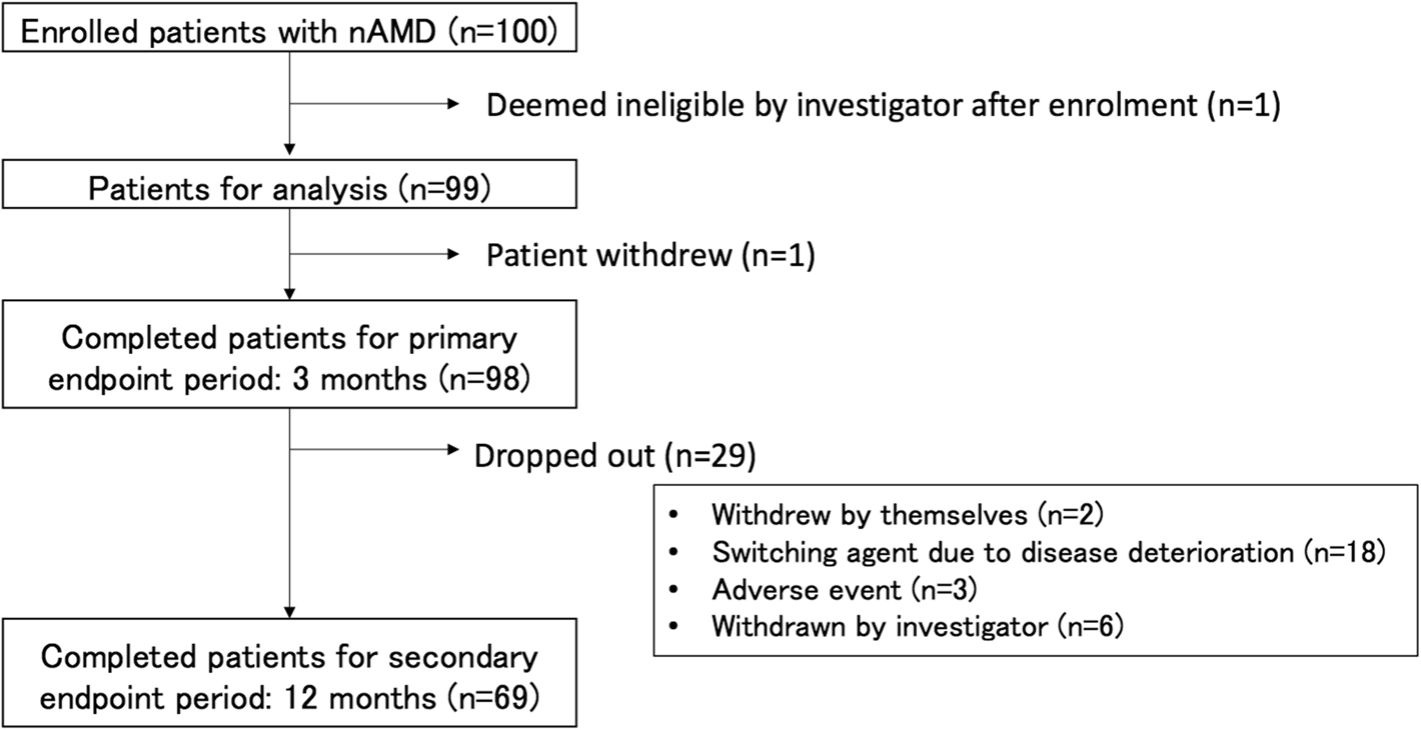
Flow of participants through the QUATRO study

**Fig. 3:**
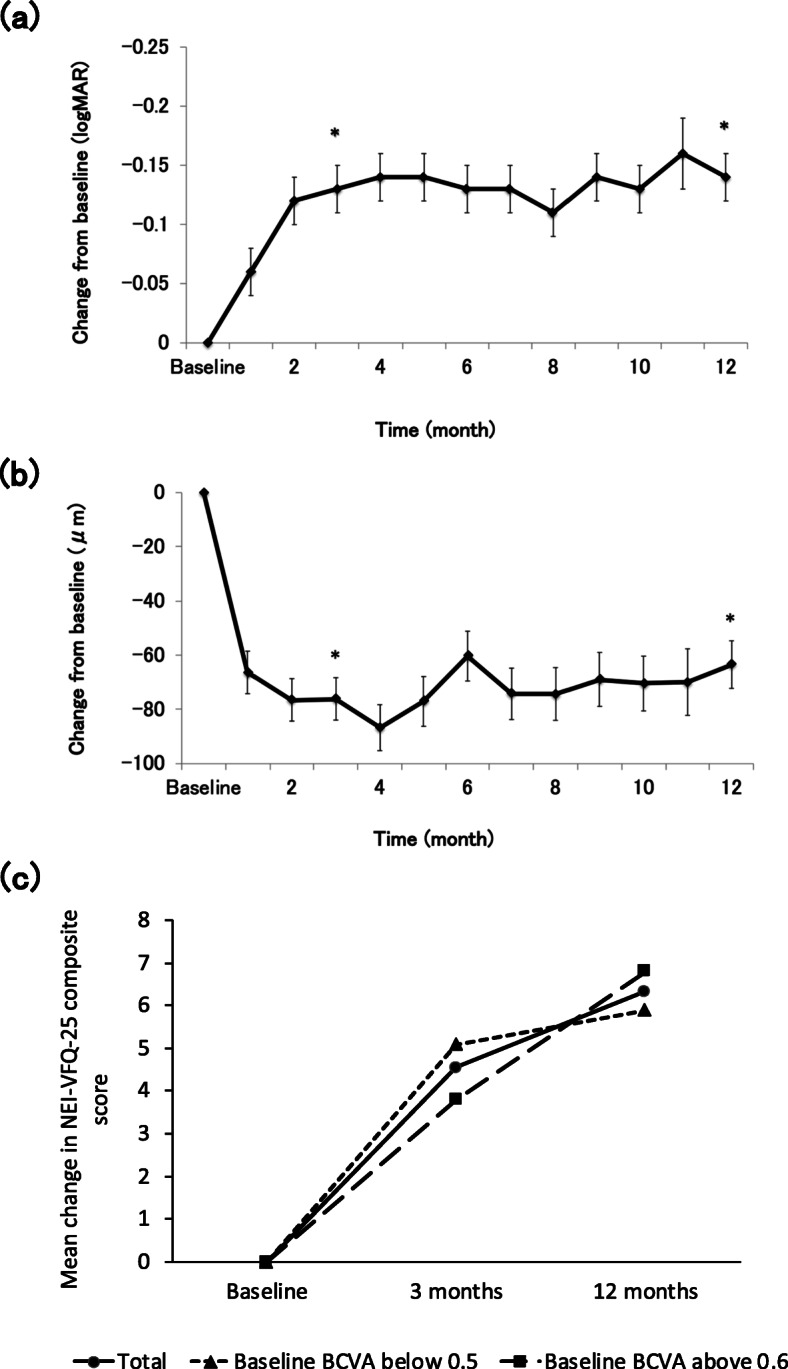
**a** The mean changes in best corrected visual acuity (BCVA) from baseline. Mean change in logMAR BCVA from baseline was − 0.13 at 3 months and − 0.14 after 1 year of treatment (*p < 0.0001). **b** The mean changes in central macular thickness (CMT). Changes in the CMT were examined by OCT for each patient. Mean change in CMT from baseline was − 76.08 μm at 3 months and −63.41 μm at 12 months (*p < 0.0001). (c) Mean changes in the NEI-VFQ-25 composite score for one year. Mean change in the NEI-VFQ-25 composite score from baseline was + 4.5 at 3 months, and + 6.3 at 12 months (*p < 0.0001)

Among the 99 patients enrolled in the study, 1 dropped out before the end of the 3-month follow-up period, and 29 more dropped out before the end of the 12-month follow-up period. Of these 30 patients who dropped out of the study, 18 dropped out after switching to another treatment agent due to disease deterioration, 3 dropped out due to adverse events, 3 withdrew themselves from the study, and 6 were withdrawn by the investigator for other reasons (Fig. 2). Mean BCVA significantly improved from 0.43 ± 0.36 logMAR at baseline to 0.28 ± 0.41 logMAR at 12 months (*p* < 0.0001). Significant improvements were also observed at 3 months (0.30 ± 0.35 LogMAR, *p* < 0.0001) (Fig. 3a).

### Anatomical outcome

The mean CMT, as measured by OCT, significantly decreased from 340.2 ± 118.3 μm at baseline to 264.3 ± 93.4 μm at 3 months (*p* < 0.0001) and to 268.4 ± 82.1 μm at 12 months (*p* < 0.0001, Fig. 3b). All patients showed exudation at baseline (intraretinal or subretinal fluid). The percentage of patients with exudative change decreased to 44.9% at 3 months and 41.2% at 12 months.

### Vision-related quality of life assessments

The mean NEI-VFQ-25 composite score was 79.4 ± 14.7 at baseline and improved to 84.1 ± 12.4 at 3 months (*n* = 98) (*P* < 0.0001) and 86.0 ± 9.2 at 12 months (*n* = 69) (*P* < 0.0001) during treatment (Fig. 3c). The mean change in the NEI-VFQ-25 composite score from baseline was significantly better in both the poor and better baseline VA groups at 3 and 12 months (mean composite score change from baseline: 5.1 in the poor group and 3.8 in the better group at 3 months, and 5.9 in the poor group and 6.8 in the better group at 12 months) (Fig. 3c). The change in the NEI-VFQ-25 composite score from baseline was associated with the logMAR VA change at 3 and 12 months (correlation coefficients = − 0.27 and − 0.31, respectively; *p* = 0.009 and 0.001, respectively). In the poor baseline VA group, the NEI-VFQ-25 score change was associated with logMAR VA change at both 3 and 12 months (correlation coefficient = − 0.33 and − 0.33, respectively; *p* = 0.02 and 0.048, respectively). In the better baseline VA group, the NEI-VFQ-25 score change was associated with logMAR VA change at 12 months, but not at 3 months (correlation coefficient = − 0.39 and − 0.27, respectively; *p* = 0.03 and 0.10, respectively). These correlations were weak but statistically significant (Table 3). For the NEI-VFQ-25 subscale scores, the mean change from baseline was ≥4 for near activities, mental health, and role difficulties in all baseline BCVA groups; as well as social functioning, dependency, and peripheral vision in the poor baseline BCVA group at 3 months. After 12 months treatment, the scores for general vision, social functioning, mental health, and role difficulties had improved by 4 or more in all baseline BCVA groups. Negative changes were identified for driving in the poor BCVA group, and driving and color vision in the better BCVA group at 3 months. After 12 months’ treatment, negative changes in the scores for ocular pain and driving were identified in the poor BCVA group, and for general health in the better BCVA group; however, no significant differences were observed from baseline (Tables 4 and 5).

**Table 3:** Correlation between NEI-VFQ-25 composite score change and logMAR BCVA change from baseline

		Correlation coefficient	*P* value
3 months (*N*=98)	Total	− 0.27	0.009
	Baseline BCVA below 0.5	−0.33	0.02
	Baseline BCVA above 0.6	− 0.27	0.10
12 months (*N*=69)	Total	−0.31	0.01
	Baseline BCVA below 0.5	−0.33	0.048
	Baseline BCVA above 0.6	−0.39	0.03

*BCVA* best-corrected visual acuity, *logMAR* logarithm of the minimal angle of resolution, *NEI-VFQ-25* 25-item National Eye Institute Visual Function Questionnaire

**Table 4:** Changes in NEI-VFQ-25 subscale scores from baseline to 3 months in each baseline vision group (*N*=98)

NEI VFQ-25 Subscale	Decimal visual acuity							
	Below 0.5				Above 0.6			
	CFB	95%CI		P value	CFB	95%CI		P value
		Lower	Upper			Lower	Upper	
Overall	5.1	2.7	7.5	0.00004	3.8	1.0	6.6	0.00729
General health	1.3	−3.5	6.2	0.58639	1.3	−4.4	7.0	0.65704
General vision	9.9	5.3	14.6	0.00005	9.2	3.8	14.7	0.00106
Ocular pain	1.1	−2.4	4.7	0.5226	2.9	−1.2	7.0	0.16917
Near activities	8.0	3.2	12.8	0.00123	7.8	2.3	13.3	0.00589
Distance activities	3.0	−0.9	6.8	0.12973	1.6	−2.9	6.1	0.4826
Social functioning	4.3	0.5	8.2	0.02752	3.5	−0.9	8.0	0.12142
Mental health	10.4	6.0	14.7	0.00001	4.5	−0.6	9.6	0.08608
Role difficulties	5.6	0.3	11.0	0.04003	5.4	−0.8	11.7	0.08844
Dependency	5.3	1.2	9.3	0.01196	3.8	−0.9	8.6	0.11354
Driving	−2.2	−8.8	4.4	0.50748	−1.5	−8.2	5.2	0.65724
Color vision	0.8	−3.3	5.0	0.69179	−0.3	−5.1	4.5	0.9098
Peripheral vision	5.5	−0.3	11.3	0.0639	1.8	−4.9	8.4	0.5975

*CFB* Change from baseline, *CI* confidence interval, *NEI-VFQ-25* 25-item National Eye Institute Visual Function Questionnaire

**Table 5:** Changes in NEI-VFQ-25 subscale scores from baseline to 12 months in each baseline vision group (*N*=69)

NEI VFQ-25 Subscale	Decimal visual acuity							
	Below 0.5				Above 0.6			
	CFB	95%CI		P value	CFB	95%CI		*P* value
		Lower	Upper			Lower	Upper	
Overall	5.9	3.2	8.5	0.00003	6.8	3.8	9.9	0.00002
General health	0.2	−5.2	5.7	0.93339	−1.7	−8.0	4.5	0.5804
General vision	9.3	4.0	14.5	0.00059	12.5	6.5	18.5	0.00006
Ocular pain	−0.1	−4.1	3.8	0.95172	0.9	−3.6	5.4	0.68663
Near activities	4.5	−0.8	9.9	0.09667	8.6	2.6	14.6	0.00561
Distance activities	2.9	−1.5	7.2	0.19592	5.7	0.7	10.6	0.02534
Social functioning	4.9	0.6	9.3	0.02557	7.0	2.1	11.9	0.00528
Mental health	16.8	11.8	21.7	0	6.1	0.5	11.7	0.03438
Role difficulties	11.4	5.3	17.5	0.00028	8.6	1.7	15.5	0.01487
Dependency	7.7	3.1	12.3	0.00116	5.0	−0.3	10.2	0.06256
Driving	−7.3	−14.9	0.3	0.05868	5.2	−2.1	12.6	0.16173
Color vision	2.1	−2.5	6.7	0.36726	3.8	−1.4	9.1	0.15108
Peripheral vision	3.9	−2.7	10.5	0.24607	6.8	−0.6	14.1	0.06986

*CFB* Change from baseline, *CI* confidence interval, *NEI-VFQ-25* 25-item National Eye Institute Visual Function Questionnaire

### Treatment satisfaction score analysis

**Fig. 4:**
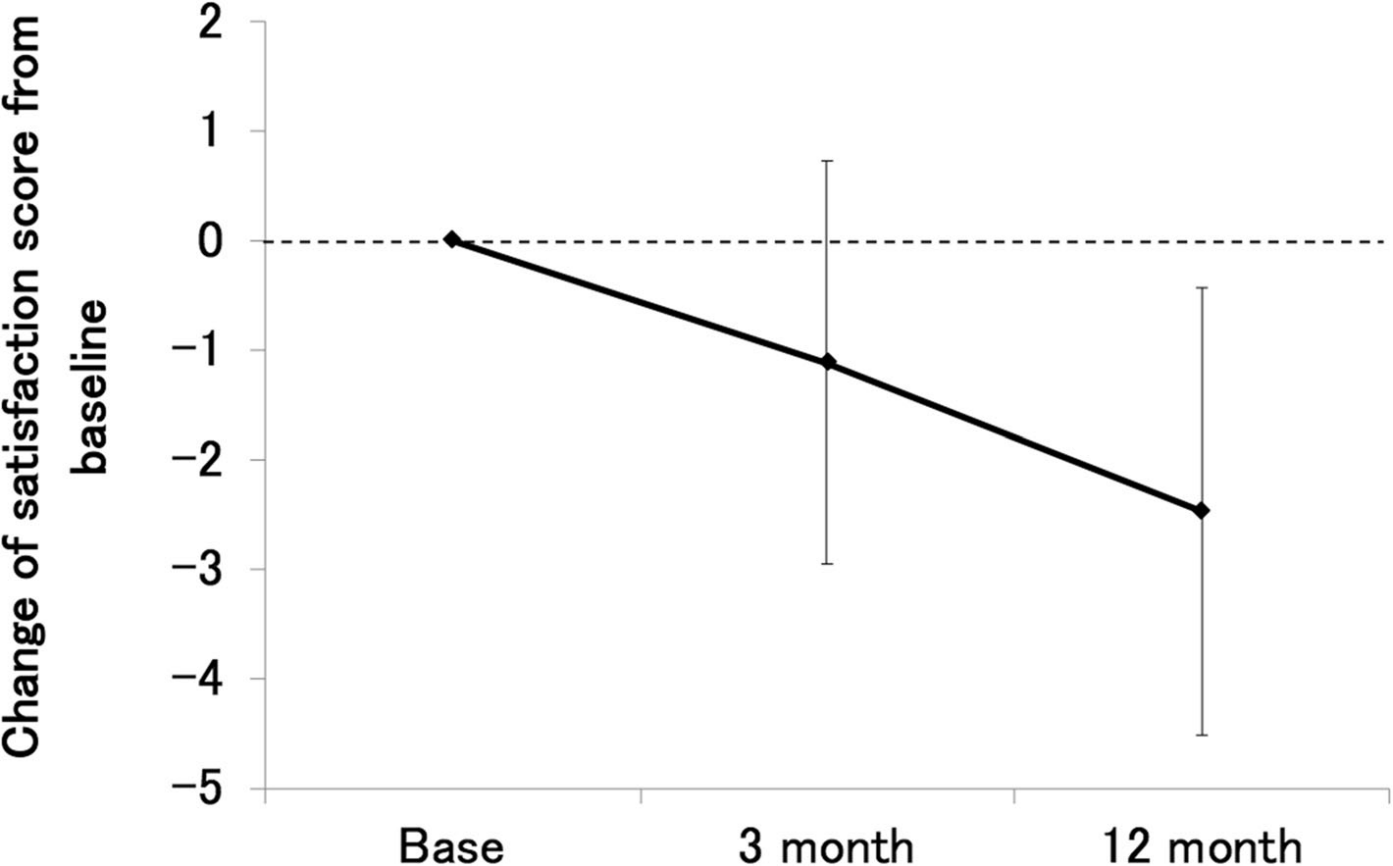
The mean changes in treatment satisfaction score for one year. Patient satisfaction questionnaire assessments were performed with a Likert scale model with five questions. Mean change in the satisfaction score from baseline was − 1.11 at 3 months (*p* = 0.54) and − 2.47 at 12 months (*p* = 0.22)

**Fig. 5:**
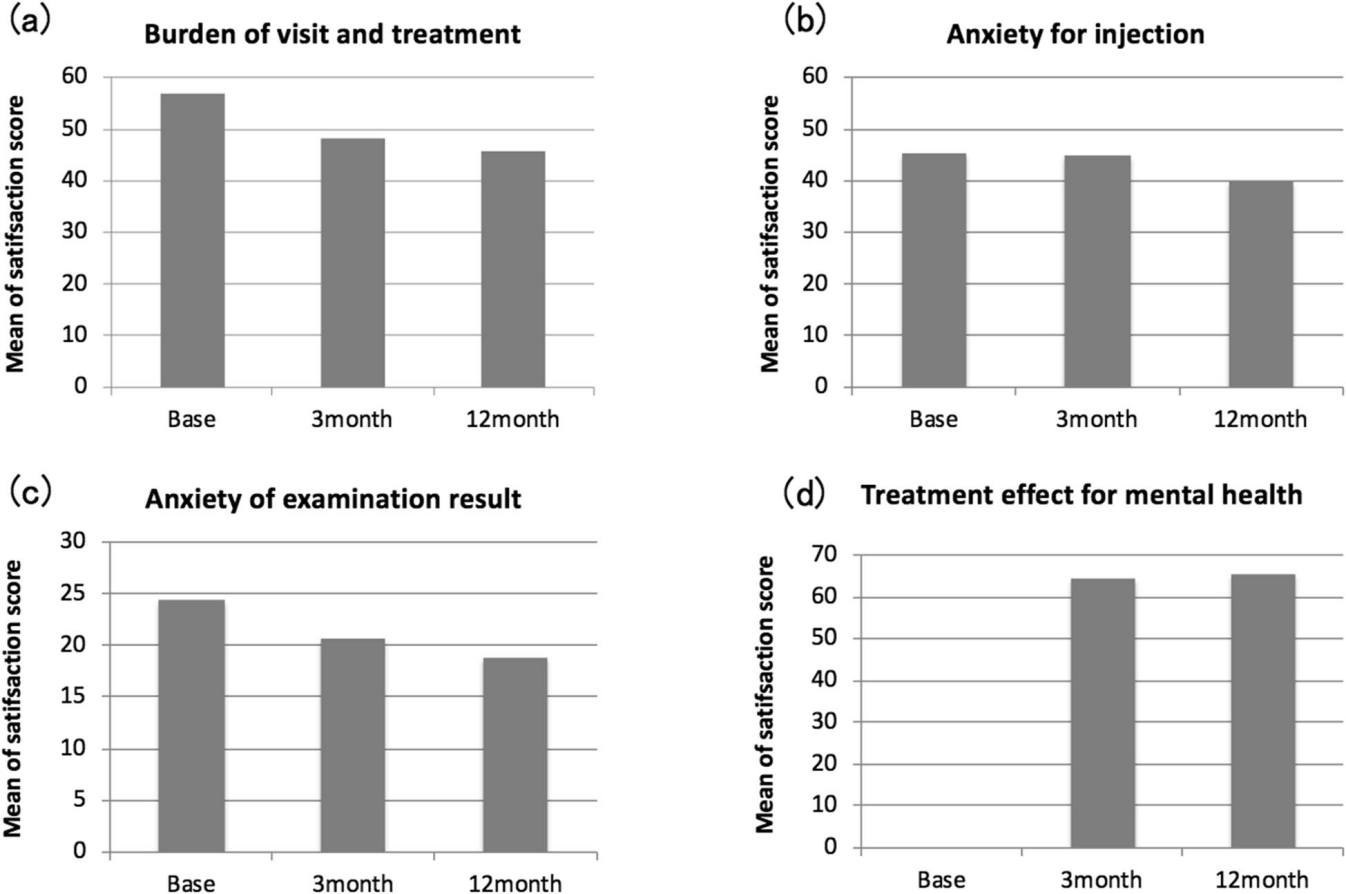
Bar graphs showing mean patient satisfaction scores in each questionnaire. **a** The burden of frequent visiting and treatment, **b** anxiety for intravitreal injection, **c** anxiety for the result of examination, **d** effect of the treatment on mental health. The satisfaction for burden of frequent visits and treatment and for anxiety of examination result which determine to receive injection **a**, **c**

The total mean satisfaction score was 61.79 ± 17.59 at baseline, 60.95 ± 16.68 at 3 months, and 58.79 ± 15.13 at 12 months. Although the mean total satisfaction scores at 3 and 12 months were lower than those at baseline, there were no significant differences from the baseline (3 months: − 1.11 ± 1.84, *p* = 0.54; 12 months: − 2.47 ± 2.04, *p* = 0.22). The subscale scores for the burdens of monthly visits and treatment were 56.79 ± 29.4 at baseline, 48.13 ± 28.3 at 3 months, and 45.81 ± 29.5 at 12 months. The subscale scores for injection anxieties were 45.37 ± 31.7 at baseline, 44.76 ± 32.7 at 3 months, and 39.97 ± 30.1 at 12 months. The scores for anxieties related to examination results were 77.78 ± 24.4 at baseline, 75.44 ± 20.6 at 3 months, and 76.21 ± 18.7 at 12 months. The scores for the effect of treatment on mental health were 64.37 ± 21.2 at 3 months and 65.6 ± 20.2 at 12 months. (Figs. 4, 5).

## Discussion

This prospective, multicenter QUATRO study revealed that intravitreous ranibizumab treatment was associated with significant improvements in QOL (composite NEI-VFQ-25 score: 4.55 at 3 months, 6.33 at 12 months) and VA (− 0.13 logMAR at 3 months, − 0.14 logMAR at 12 months) in Japanese patients with nAMD. The significant correlation between the mean changes from baseline for the NEI-VFQ-25 composite score and logMAR VA was observed in all patients at 3 and 12 months. Major clinical trials have similarly shown an improvement in vision-related quality of life for patients with nAMD receiving either intravitreal ranibizumab injection (IVR) or intravitreal aflibercept injection (IVA) treatment [19, 20, 22]. This study revealed significant correlations between the NEI-VFQ-25 composite score changes and logMAR VA changes in both poor and better baseline VA subgroups at 12 months. However, at 3 months, the two parameters were significantly correlated only in the poor baseline VA group; the reason for this is possibly because the baseline VA in this study was better than that in clinical studies. Moreover, the ceiling effect may also explain why a gain in vision has a greater impact on the QOL of patients with poor vision than those with good vision. The BCVA inclusion criteria for major clinical studies are almost equal to those of the poor BCVA group in this study (0.05 ≤ decimal VA < 0.6). Moreover, the baseline NEI-VFQ-25 composite score in our study (79.4) was higher than that in randomized clinical studies (68–72) [19, 20, 22]. The MARINA and ANCHOR studies, which are both major clinical studies on ranibizumab treatment in patients with nAMD, revealed that a 15-letter change in BCVA over 12 months corresponded to a 4–6-point increase in the NEI-VFQ-25 composite score from a mean baseline score of 69.3 and 69.9 [25]. In this study, the mean change in the NEI-VFQ-25 composite score from baseline was also similar (5.9 points in the poor BCVA group and 6.8 points in the better BCVA group at 12 months). The mean changes in the NEI-VFQ-25 subscale scores (6 of 12 subscales in the poor VA group and 2 of 12 in the better VA group at 3 months, and 4 of 12 in the poor VA group and 6 of 12 in the better VA group at 12 months) showed a significant (*P* < 0.05) and gradual improvement. We also regarded a change of ≥4 in the NEI-VFQ-25 composite and subscale scores from baseline as clinically significant, as published previously [21]. This threshold was based on a published study in which a BCVA change of 15 letters corresponded to a NEI-VFQ-25 composite score change of 4 in patients with nAMD [25]. This score was regarded as the minimally important difference (MID) for the NEI-VFQ-25 score in nAMD [26]. The mean number of subscales showing NEI-VFQ-25 subscale score changes ≥ 4 in our study were 6 out of 12 in the poor BCVA group and 4 out of 12 in the better BCVA group at 3 months, and 6 out of 12 in the poor BCVA group and 9 out of 12 in the better BCVA group. Clinically significant improvements in many QOL subscales were observed, especially in the better BCVA group. These results indicated that the vision-related QOL of Japanese patients with nAMD improved with ranibizumab treatment, regardless of whether the baseline BCVA was poor or better. However, clinically significant improvements (subscale score changes < 4 points) were not found in the following subscales: ocular pain, distance activities, driving, and color vision at 3 months in both the poor and better baseline BCVA groups; and ocular pain, distance activities, and color vision at 12 months in both the poor and better baseline BCVA groups. This result is similar to the findings of other clinical studies, with color vision, ocular pain, and general health not showing clinically significant improvements in the MARINA study [19]. Interestingly, there were discrepancies in the subscale score changes for driving between the poor and better baseline BCVA groups at 12 months (+ 5.2-point gain in the better BCVA group and − 7.3-point loss in the poor BCVA group). The reason for this discrepancy could be that most patients with better VA exhibited a clinical improvement sufficient to allow driving after 12 months of ranibizumab treatment, whereas most of the patients in the poor VA group could not reach the VA standard for driving. These results indicate the importance of initiating treatment as early as possible, even if the patient’s VA is good.

A treatment satisfaction survey was performed in this QUATRO study. Patient satisfaction was statistically stable, but the total satisfaction score decreased gradually at 3 months, and 12 months (3 months, *P* = 0.54; 12 months, *P* = 0.22). In the subscale analysis, satisfaction scores for “anxiety for injection,” and “treatment effect for mental health” were almost stable, whereas scores for “burden for visiting and treatment” and “anxiety of examination result” declined. Based on this survey, we speculate that patients were especially dissatisfied with the frequent visits and treatment burden, although their VA and vision-related QOL had improved in a real-life setting. We also speculate that participants were stressed by not knowing whether or not they were going to be treated on that day. Similar outcomes were observed in some treatment satisfaction surveys for patients with nAMD in a real-life setting. Gohil et al. reported that treatment satisfaction for nAMD was determined by the notion of being reviewed and injected routinely over a long period rather than the actual change in VA due to the treatment [27]. Recently, many physicians prefer proactive treatment (fixed dosing, treat-and-extend [TAE]) to reactive treatment (PRN) to avoid VA decline in the maintenance phase after the three loading injections for nAMD. TAE has been shown to be a good way of maintaining the patients’ vision gains by treatment and reducing their burden [15, 28, 29]. We also considered the burdens for the patients’ companions, caregivers, and families. Ranibizumab PRN therapy is linked to significant caregiver burden, and it has been reported that both the disease effect and treatment frequency contribute to the overall burden [30]. Hanemoto et al. compared the burden for caregivers and patients with nAMD by TAE or PRN treatment regimen. Caregivers of patients receiving TAE had a lower productivity loss associated with accompanying patients to the hospital than those receiving PRN; thus leading them to conclude that TAE was associated with a lower caregiver burden, time and costs included [31]. Joko et al. investigated patient preferences for anti-VEGF therapy for the treatment of nAMD using trade-offs between dosing regimens. They reported that patients were willing to agree to an increase in injections, from three to approximately eight, in 12 months to improve the chance of 1-year VA melioration from 25 to 40%. Between the dosing regimens, patients tended to prefer TAE due to its higher chance of 2-year VA maintenance [32]. In the future, it will be necessary to consider the treatment burden and satisfaction of patients to maintain their vision and vision-related quality of life.

This study had several limitations. The study period of 1 year could be considered relatively short and the number of questionnaires for the satisfaction survey was small. In this study, we focused on the clinical outcomes of the treated eye and did not collect BCVA data from the other eye. Therefore, we could not consider the untreated eye’s BCVA in our analyses. However, this was a prospective, multicenter study, which used Japanese patients with nAMD, rendering our data robust.

## Conclusions

In conclusion, this prospective, multicenter, open-label study of patients with nAMD conducted in Japan examining the differences in vision-related QOL according to baseline VA by ranibizumab therapy revealed equal improvement in QOL, which correlated with the VA improvement over a 1-year treatment period. Several key NEI-VFQ-25 subscale scores also exceeded the MID threshold. We believe that therapeutic intervention should be performed as early as possible. The patients’ satisfaction with the treatment was stable over 1 year but did not increase. Regardless, future research needs to have longer follow-up periods and a comparison of treatment regimens.

## Data Availability

Data are available from Yuji Oshima (yuji@eye.med.kyushu-u.ac.jp) for researchers who meet the criteria for access to confidential data.

## Abbreviations

AMD: Age-related macular degeneration
BCVA: Best-corrected visual acuity
CMT: Central macular thickness
logMAR: Logarithm of the minimal angle of resolution
CNV: Choroidal neovascularization
PCV: Polypoidal choroidal vasculopathy
RAP: Retinal angiomatous proliferation
VEGF: Vascular endothelial growth factor
NEI-VFQ-25: 25-item National Eye Institute Visual Function Questionnaire
CFB: Change from baseline
CI: Confidence interval
OCT: Optical coherence tomography
FA: Fluorescein angiography
IA: Indocyanine green angiography
GLD: Greatest linear dimension

## References

[1] Fine SL, Berger JW, Maguire MG, Ho AC (2000). Age-related macular degeneration. N Engl J Med.

[2] Francis PJ, Klein ML (2011). Update on the role of genetics in the onset of age-related macular degeneration. Clin Ophthalmol.

[3] Rosenfeld PJ, Brown DM, Heier JS, Boyer DS, Kaiser PK, Chung CY (2006). Ranibizumab for neovascular age-related macular degeneration. N Engl J Med.

[4] Schmidt-Erfurth U, Kaiser PK, Korobelnik J-F, Brown DM, Chong V, Nguyen QD (2014). Intravitreal aflibercept injection for neovascular age-related macular degeneration: ninety-six-week results of the VIEW studies. Ophthalmology..

[5] Tano Y, Ohji M, EXTEND-I Study Group (2010). EXTEND-I: safety and efficacy of ranibizumab in Japanese patients with subfoveal choroidal neovascularization secondary to age-related macular degeneration. Acta Ophthalmol.

[6] Takahashi K, Ogura Y, Ishibashi T, Shiraga F, Yuzawa M (2012). Treatment guidelines for age-related macular degeneration. Nihon Ganka Gakkai Zasshi.

[7] Brown DM, Kaiser PK, Michels M, Soubrane G, Heier JS, Kim RY (2006). Ranibizumab versus verteporfin for neovascular age-related macular degeneration. N Engl J Med.

[8] Brown DM, Heier JS, Ciulla T, Benz M, Abraham P, Yancopoulos G (2011). Primary endpoint results of a phase II study of vascular endothelial growth factor trap-eye in wet age-related macular degeneration. Ophthalmology..

[9] Toalster N, Russell M, Ng P (2013). A 12-month prospective trial of inject and extend regimen for ranibizumab treatment of age-related macular degeneration. Retina..

[10] Sultan MB, Zhou D, Loftus J, Dombi T, Ice KS (2011). Macugen 1013 Study Group. A phase 2/3, multicenter, randomized, double-masked, 2-year trial of pegaptanib sodium for the treatment of diabetic macular edema. Ophthalmology.

[11] Heier JS, Brown DM, Chong V, Korobelnik J-F, Kaiser PK, Nguyen QD (2012). Intravitreal aflibercept (VEGF trap-eye) in wet age-related macular degeneration. Ophthalmology..

[12] Rofagha S, Bhisitkul RB, Boyer DS, Sadda SR, Zhang K, SEVEN-UP Study Group (2013). Seven-year outcomes in ranibizumab-treated patients in ANCHOR, MARINA, and HORIZON: a multicenter cohort study (SEVEN-UP). Ophthalmology..

[13] DeCroos FC, Reed D, Adam MK, Salz D, Gupta OP, Ho AC (2017). Treat-and-extend therapy using Aflibercept for Neovascular age-related macular degeneration: a prospective clinical trial. Am J Ophthalmol.

[14] Ohnaka M, Nagai Y, Sho K, Miki K, Kimura M, Chihara T (2017). A modified treat-and-extend regimen of aflibercept for treatment-naïve patients with neovascular age-related macular degeneration. Graefes Arch Clin Exp Ophthalmol.

[15] Ohji M, Takahashi K, Okada AA, Kobayashi M, Matsuda Y, Terano Y (2020). Efficacy and safety of Intravitreal Aflibercept treat-and-extend regimens in exudative age-related macular degeneration: 52- and 96-week findings from ALTAIR : a randomized controlled trial. Adv Ther.

[16] Mangione CM, Gutierrez PR, Lowe G, Orav EJ, Seddon JM (1999). Influence of age-related maculopathy on visual functioning and health-related quality of life. Am J Ophthalmol.

[17] Suzukamo Y, Oshika T, Yuzawa M, Tokuda Y, Tomidokoro A, Oki K (2005). Psychometric properties of the 25-item National eye Institute visual function questionnaire (NEI VFQ-25), Japanese version. Health Qual Life Outcomes.

[18] Bressler NM, Chang TS, Fine JT, Dolan CM, Ward J (2009). Anti-VEGF antibody for the treatment of predominantly classic Choroidal neovascularization in age-related macular degeneration (ANCHOR) research group. Improved vision-related function after ranibizumab vs photodynamic theraphy: a randomized clinical trial. Arch Ophthalmol.

[19] Chang TS, Bressler NM, Fine JT, Dolan CM, Ward J, Klesert TR (2007). Improved vision-related function after ranibizumab treatment of neovascular age-related macular degeneration: results of a randomized clinical trial. Arch Ophthalmol.

[20] Yuzawa M, Fujita K, Wittrup-Jensen KU, Norenberg C, Zeitz O, Adachi K (2015). Improvement in vision-related function with intravitreal aflibercept: data from phase 3 studies in wet age-related macular degeneration. Ophthalmology..

[21] Gomi F, Migita H, Sakaguchi T, Okada H, Sugawara T, Hikichi Y (2019). Vision-related quality of life in Japanese patients with wet age-related macular degeneration treated with intravitreal aflibercept in a real-world setting. Jpn J Ophthalmol.

[22] Bressler NM, Chang TS, Suñer IJ, Fine JT, Dolan CM, Ward J, et al. Vision-related function after ranibizumab treatment by better- or worse-seeing eye: clinical trial results from MARINA and ANCHOR. Ophthalmology. 2010;117:747–756.e4. 10.1016/j.ophtha.2009.09.002 20189654

[23] Oshima Y, Kimoto K, Yoshida N, Fujisawa K, Sonoda S, Kubota T (2017). One-year outcomes following Intravitreal Aflibercept for Polypoidal Choroidal vasculopathy in Japanese patients: the APOLLO study. Ophthalmologica..

[24] Wada I, Oshima Y, Shiose S, Kano K, Nakao S, Kaizu Y (2019). Five-year treatment outcomes following intravitreal ranibizumab injections for neovascular age-related macular degeneration in Japanese patients. Graefes Arch Clin Exp Ophthalmol.

[25] Suñer IJ, Kokame GT, Yu E, Ward J, Dolan C, Bressler NM (2009). Responsiveness of NEI VFQ-25 to changes in visual acuity in neovascular AMD: validation studies from two phase 3 clinical trials. Invest Ophthalmol Vis Sci.

[26] Yuzawa M, Fujita K, Tanaka E, Wang ECY (2013). Assessing quality of life in the treatment of patients with age-related macular degeneration: clinical research findings and recommendations for clinical practice. Clin Ophthalmol.

[27] Gohil R, Crosby-Nwaobi R, Forbes A, Burton BJ, Hykin P, Sivaprasad S (2016). Treatment satisfaction of patients undergoing ranibizumab therapy for neovascular age-related macular degeneration in a real-life setting. Patient Prefer Adher.

[28] Wykoff CC, Ou WC, Brown DM, Croft DE, Wang R, Payne JF (2017). Randomized trial of treat-and-extend versus monthly dosing for Neovascular age-related macular degeneration: 2-year results of the TREX-AMD study. Ophthalmol Retina.

[29] Ohji M, Lanzetta P, Korobelnik J-F, Wojciechowski P, Taieb V, Deschaseaux C (2020). Efficacy and treatment burden of Intravitreal Aflibercept versus Intravitreal Ranibizumab treat-and-extend regimens at 2 years: network meta-analysis incorporating individual patient data meta-regression and matching-adjusted indirect comparison. Adv Ther.

[30] Gohil R, Crosby-Nwaobi R, Forbes A, Burton B, Hykin P, Sivaprasad S (2015). Caregiver burden in patients receiving Ranibizumab therapy for Neovascular age related macular degeneration. PLoS One.

[31] Hanemoto T, Hikichi Y, Kikuchi N, Kozawa T (2017). The impact of different anti-vascular endothelial growth factor treatment regimens on reducing burden for caregivers and patients with wet age-related macular degeneration in a single-center real-world Japanese setting. PLoS One.

[32] Joko T, Nagai Y, Mori R, Tanaka K, Oshima Y, Hikichi Y (2020). Patient preferences for anti-vascular endothelial growth factor treatment for wet age-related macular degeneration in Japan: a discrete choice experiment. Patient Prefer Adherence.

